# Mining the candidate transcription factors modulating dendrobine biosynthesis under phosphate deficiency in *Dendrobium officinale* Kimura & Migo

**DOI:** 10.3389/fpls.2026.1784768

**Published:** 2026-03-03

**Authors:** Siqi Gui, Jiening Wu, Yifei Shi, Chao Zhuang, Junjie Zhong, Xiaowei Zou, Hui Du, Wei Zhou

**Affiliations:** School of Pharmaceutical Sciences, The First Affiliated Hospital of Zhejiang Chinese Medical University, Hangzhou, China

**Keywords:** dendrobine biosynthesis, *Dendrobium officinale* Kimura & Migo, phosphate-deficiency stress, transcription factors, transcriptome

## Abstract

Phosphorus is integral to energy transfer and structural integrity in plants, which plays a significant role in regulating secondary metabolism. Notably, low phosphorus (LP) stress significantly improves dendrobine content in *Dendrobium officinale*, yet the molecular basis for this induction remains unclear. This study employed transcriptomic analysis to identify the differentially expressed genes (DEGs) related to the dendrobine biosynthesis under LP stress in *D. officinale*. 1,713, 222, 488, and 174 DEGs were up-regulated among the different phosphorus treatment groups, including the HP (high phosphorus) vs TP (total phosphorus), MP (medium phosphorus) vs TP, LP (low phosphorus) vs TP and NP (no phosphorus) vs. TP, respectively. In contrast, 1,855, 195, 432, and 120 DEGs exhibited a down-regulated expression pattern between each of them, respectively. Gene annotation in public datasets revealed that the DEGs related to phosphate transporter and alkaloid biosynthesis were enriched in *D. officinale*. By co-expression analysis, 10 phosphorus transport-related transcription factors (TFs) and 21 TFs associated with dendrobine biosynthesis were mined from the *D. officinale* transcriptome. These above findings provide many candidate TFs related to dendrobines biosynthesis and new insights into dissecting the potential molecular mechanism on regulating dendrobine biosynthesis under LP stress in *D. officinale*.

## Introduction

The medicinal plant *Dendrobium officinale* Kimura & Migo (commonly known as “Tiepi Shihu” in China) is a perennial epiphytic herb of the Orchidaceae family and the Dendrobium genus. It is a rare and valuable medicinal plant in traditional Chinese medicine (Cakova et al., 2017; Wang, 2021; Li et al., 2025). *D. officinale* contains various bioactive compounds, with polysaccharides and dendrobines as the major criteria for quality assessment in Chinese pharmacopoeia (Cakova et al., 2017; Wang, 2021; Li et al., 2024). Among them, the dendrobines present in *D. officinale* exhibit a variety of therapeutic effects against cancer, cardiovascular diseases, and gastrointestinal disorders. Additionally, they possess analgesic and antipyretic properties (Inubushi et al., 1963; Li et al., 2017; Liu et al., 2020b; Zhang et al., 2022; Huang et al., 2023; Okoro et al., 2023; Yang et al., 2023; Guo et al., 2024; Huang et al., 2024). Many studies have validated that environmental factors significantly influence dendrobine levels in *D. officinale*. Notably, phosphorus has been identified as a confirmed regulator of secondary metabolite biosynthesis in medicinal plants (Zuo et al., 2020; Chen et al., 2025).

Dendrobine is a picrotoxane-type sesquiterpene alkaloid whose biosynthesis follows a typical sesquiterpene pathway (Gong et al., 2021; Li et al., 2017). Its building blocks, isopentenyl diphosphate (IPP) and dimethylallyl diphosphate (DMAPP), are derived from both the mevalonate (MVA) pathway (starting from acetyl−CoA) and the methylerythritol phosphate (MEP) pathway (starting from glyceraldehyde−3−phosphate and pyruvate) (Shi et al., 2016; Huang et al., 2023). IPP and DMAPP are then further converted by the enzymes of geranyl diphosphate synthase (GPPS) and farnesyl diphosphate synthase (FPPS) to generate farnesyl pyrophosphate (FPP). In the downstream pathway of dendrobine biosynthesis, FPP is used as the substrate and undergoes two catalytic reactions by multiple terpene synthases (TPSs) to form the molecular skeleton of copacamphane (Gong et al., 2021). This skeleton is then subjected to multiple catalytic reactions by cytochrome P450 (CYP450) enzymes (including CYP71D55, CYP94C1 and other unknown CYP450 members) to produce picrotoxane-lactone, which is the basic skeleton molecule of dendrobine (Li et al., 2017). This basic skeleton molecule subsequently undergoes amination, methylation, and cyclization-decarboxylation reactions to ultimately yield dendrobine.

Phosphorus is a key nutrient element that regulates both primary and secondary metabolism in plants. Notably, phosphorus has been confirmed as a regulatory factor for the biosynthesis of secondary metabolites in medicinal plants. Low-phosphorus (LP) stress can promote the biosynthesis of phenolic acids in *Salvia miltiorrhiza* (Hao et al., 2020) and enhance the accumulation of tanshinones in its hairy roots (Zheng et al., 2023), while it inhibits alkaloid biosynthesis in *Anisodus tanguticus* (Zhang et al., 2025). Similarly, in *D. officinale*, applying LP stress within a comparable concentration range (typically spanning from deficiency to sufficiency, e.g., 0 to 2.5 mM) is known to increase alkaloid content (Liu et al., 2021). However, the transcriptional regulatory network, especially the key transcription factors, remains largely unexplored in *D. officinale*.

To dissect such complex regulatory mechanisms, identifying key transcription factors (TFs) is essential. In medicinal plants, transcription factors play a fundamental role in regulating secondary metabolism by controlling the transcription of biosynthetic genes. In *Camptotheca acuminata*, *OpWRKY1* directly downregulates the expression of cytochrome P450 reductase (CYP) gene in hairy roots, thereby inhibiting the camptothecin (CPT) biosynthesis (Xu et al., 2020). *OpERF2*, when suppressed via RNA interference (RNAi), leads to a reduced expression pattern of those genes in MEP and secologanin-strictosidine pathways, indicating that *OpERF2* promotes CPT biosynthesis (Udomsom et al., 2016). In *Catharanthus roseus*, *CrWRKY1* positively regulates the biosynthesis of bisindole alkaloids by activating the *TDC* gene through binding to the W-box element in its promoter, while simultaneously suppressing the expression of several terpenoid indole alkaloids (TIA) biosynthesis activators, such as *ORCA2*, *ORCA3*, and *CrMYC2* (Suttipanta et al., 2011). In *Anisodus acutangulus*, *AaWRKY11* activates the expression of hyoscyamine 6β-hydroxylase (H6H1) gene, leading to tropane alkaloids accumulation in *A. acutangulus* (Zhou et al., 2024). These cases establish TFs as central molecular switches connecting environmental cues to metabolic outputs. Nevertheless, the specific TFs that mediate the response to LP stress and concurrently regulate dendrobine biosynthesis in *D. officinale* have not to be systematically identified and characterized.

To address this, the present study employed a phosphorus gradient (0–2.5 mM KH_2_PO_4_) centered around the standard MS medium concentration. An integrated approach of transcriptome sequencing and gene co−expression network analysis was implemented. First, transcription factors (TFs) responsive to low−phosphorus (LP) stress were identified. Subsequently, candidate TFs potentially coregulating phosphorus−signaling adaptation and dendrobine biosynthesis were mined. Finally, the expression patterns of key candidates were validated via qRT−PCR detection. This work provides a transcriptional regulatory framework for LP−enhanced dendrobine accumulation and offers genetic targets for breeding phosphorus−efficient *D. officinale* varieties.

## Materials and methods

### Experimental design

For RNA-sequencing and dendrobine quantification, ten-month-old sterile *D. officinale* seedlings were used. Seedlings were grouped (ten per replicate) and treated with a gradient of inorganic phosphorus (Pi) concentrations. This was achieved by supplementing phosphorus-free Murashige and Skoog (MS) medium with KH_2_PO_4_ to the following final concentrations: 1.25 mM (total phosphorus, TP), 2.5 mM (high phosphorus, HP), 0.625 mM (medium phosphorus, MP), 0.0625 mM (low phosphorus, LP), and 0 mM (no phosphorus, NP). The TP treatment group served as the mock group. All plants were cultivated in a greenhouse for 40 days under controlled conditions: 25 °C, 60% relative humidity, and a 12-h light (200 μmol·m^-^²·s^-^¹)/12-h dark photoperiod. After the treatment period, the seedlings were harvested for subsequent transcriptome analysis and dendrobine content measurement (Ren et al., 2022; Liu et al., 2022). All collected samples were snap-frozen and stored at -80 °C, with three biological replicates used for all experiments.

### Determination of dendrobine content

Total dendrobine content was determined according to a previous method (Wang et al., 2016). Each 0.5 g sample of dried stem was finely powdered and poured into a distillation flask. The sample was moistened with 10% ammonia solution and kept for 30 minutes. Subsequently, 10 mL of chloroform was added into sample, and the distillation flask was initially weighed. Next, the dendrobine was extracted for 2 h with a condensation reflux device at 65 °C. After cooling, an appropriate volume of chloroform was added into the crude extract to restore the total weight to its initial value. The crude extracts were collected and filtered. Finally, 2 mL of filtered extract was mixed with 8 mL of chloroform to prepare the final tested solution, which was used to measure the absorbance value at a wavelength of 620 nm using an ultraviolet-visible spectrophotometry device. The total dendrobine content in each *D. officinale* sample was calculated based on the standard curve.

### RNA extraction and Illumina sequencing

*D. officinale* seedlings subjected to five Pi treatments were collected. Following extraction of total RNA with TRIzol^®^ Reagent and quality confirmation (Nanodrop 2000 and gel electrophoresis), mRNA was purified using Oligo (dT) magnetic beads. Subsequently, a library was prepared from 1 µg of mRNA. Paired-end sequencing was conducted on the Illumina NovaSeq X Plus platform by Shanghai Majorbio Bio-pharm Technology Co., Ltd (Zhang et al., 2024).

### *De novo* assembly and functional annotation

To ensure data quality, raw sequencing reads were processed with fastp (Version 1.0.1) with default parameters. to remove adapter sequences and filter low-quality reads (Chen et al., 2018). The resulting clean reads were aligned to the reference genome (ASM160598v2) in orientation mode using HISAT2 software (Version 2.2.1) to generate mapped reads for subsequent transcript assembly and expression level calculation (Kim et al., 2015; Zhang et al., 2016). Functional annotation of the assembled transcripts was then performed by scanning six public databases (EggNOG, Swiss-Prot, GO, Pfam, KEGG, and NR) with Diamond and HMMER, using an E-value cutoff of 1×10^-5^ (Cheng et al., 2025a). The highest-scoring annotation for each gene was retained to compile the final annotation list.

### Identification of DEGs

Differentially expressed genes (DEGs) were identified between the mock (TP) group and each treatment group (HP, MP, LP, and NP). Expression levels were quantified as FPKM (Fragments Per Kilobase of transcript per Million mapped reads) using RSEM software (Version 1.3.3) (Pertea et al., 2015). Differential expression analysis was performed with DESeq2. Genes with an absolute log_2_ fold change (|log_2_FC|)≥1 and a false discovery rate (FDR) < 0.05 were identified as significant DEGs (Love et al., 2014).

### Mining the candidate TFs by weighted gene co-expression network analysis

To identify candidate transcription factors involved in modulating dendrobine biosynthesis in response to low-phosphorus (LP) stress, expression correlation analysis was performed. Spearman’s rank correlation was used to calculate pairwise associations between genes, with an absolute correlation coefficient threshold of |r| ≥ 0.9. Correlations were considered significant if the adjusted P-value (padj) was < 0.05 after Benjamini–Hochberg correction. The expression profile of candidate TFs was presented in heatmaps generated using the Cytoscape software.

### Quantitative real-time quantitative PCR

For experimental verification of transcriptome data, qRT−PCR assays were performed. First−strand cDNA was generated from 100 ng total RNA. The Applied Biosystems 7500 system and *Taq* Pro Universal SYBR qPCR Master Mix (Vazyme, China) were employed, with primers listed in **Supplementary Table S1**. The 10−µL reaction volume comprised 1 µL cDNA, 0.5 µL of each primer, 5 µL 2× Master Mix, and 3 µL ddH_2_O. Using the 2^−ΔΔCT^ method and *DoActin* as the reference gene, relative expression of candidate gene was derived. All data were obtained from 3 independent biological replicates.

## Results

### Low Pi improves dendrobine biosynthesis in *D. officinale*

**Figure 1:**
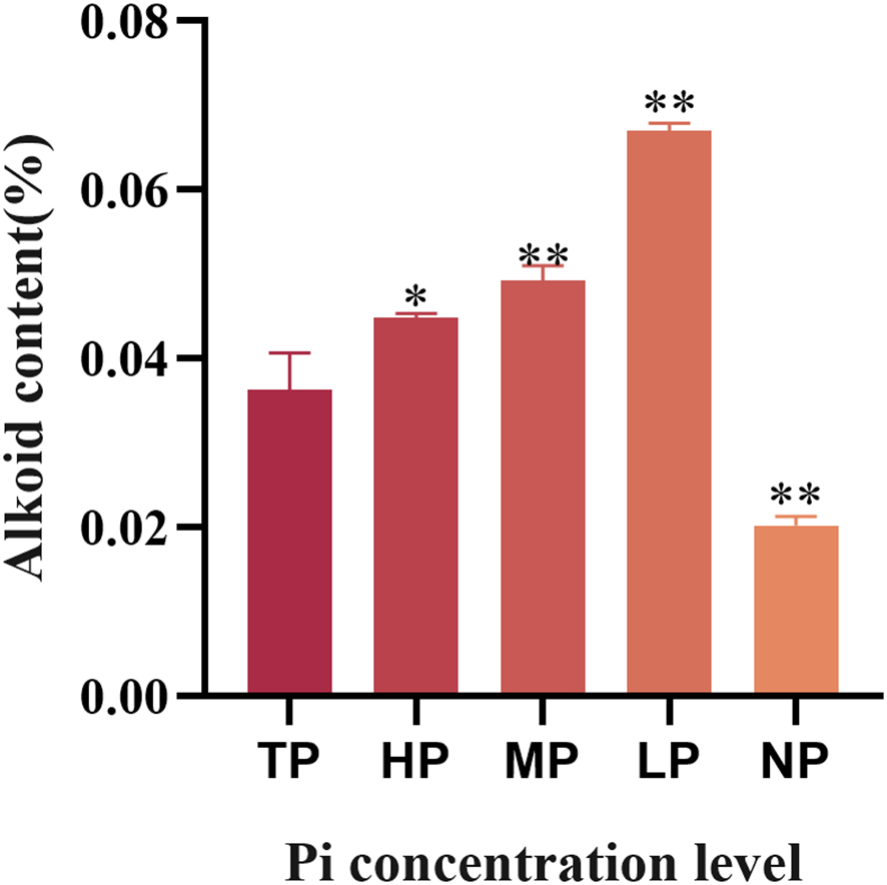
Effects of phosphorus deficiency on the dendrobine accumulation in *D. officinale.* TP, HP, MP, LP and NP denote total, high, medium, low, and no phosphorus, respectively. Data are presented as mean ± SD (*n* = 3). Significant differences were determined by Student’s *t*-test (*P < 0.05; **P < 0.01).

To examine how low Pi affects dendrobine biosynthesis in *D. officinale*, the total content of dendrobine in each *D. officinale* plantlet treated with five different Pi concentrations was detected by spectrophotometry, respectively. Under LP treatment, the dendrobine content elevates to 0.07% of the total dry weight, representing a 1.85-fold increase relative to the Mock control (TP) (**Figure 1**). It implies that low Pi can improve the dendrobine biosynthesis in *D. officinale*.

### Transcriptome sequencing and *de novo* assembly of *D. officinale* transcripts

Through transcriptome sequencing, a total of 731 million raw reads were obtained from the 15 samples, with the average clean data more than 6.19 Gb (**Supplementary Table S2**). The GC content of each sample ranged from 44.93% to 46.59%, and the Q30 of clean reads was greater than 96.5% (**Table 1**). The high-quality reads from every sample were aligned against the *D. officinale* reference genome (ASM160598v2), yielding alignment rates between 86.75% and 89.87% (**Supplementary Table S3**). Evaluation of sequencing coverage and transcript length distribution indicates that the quality of assembled transcriptome data are sufficient for further data mining (**Supplementary Figure S1**).

**Table 1:** Summary of the *D. officinale* transcriptome.

Sample	Raw reads	Clean reads	Clean bases	Q30 (%)	GC content (%)
TP_1	49,300,714	48,913,774	7,306,208,623	96.74	45.35
TP_2	41,807,246	41,474,796	6,202,588,927	96.62	45.2
TP_3	46,143,358	45,731,912	6,824,993,444	96.67	45.16
HP_1	47,627,626	47,251,262	7,047,670,065	96.71	44.93
HP_2	45,157,276	44,782,028	6,691,124,939	96.5	45
HP_3	41,758,584	41,427,312	6,189,244,159	96.52	45.04
MP_1	72,159,922	71,501,996	10,663,062,508	96.63	45.19
MP_2	74,014,092	73,419,776	10,969,199,898	96.62	45.16
MP_3	47,542,948	47,016,608	6,999,118,657	96.62	46.59
LP_1	43,111,822	42,733,908	6,380,666,647	96.67	45.12
LP_2	42,019,830	41,663,600	6,217,752,257	96.64	45.24
LP_3	43,423,554	43040112	6,427,049,986	96.66	45.24
NP_1	47,766,948	47,381,206	7,079,937,680	96.69	45.4
NP_2	44,193,734	43,807,358	6,565,192,759	96.69	44.97
NP_3	45,707,294	45,354,450	6,804,259,651	96.55	45.07
Average	48,782,330	48,366,673	7,224,538,013	96.63	45.24

### Transcriptome sequencing and gene expression analysis

**Figure 2:**
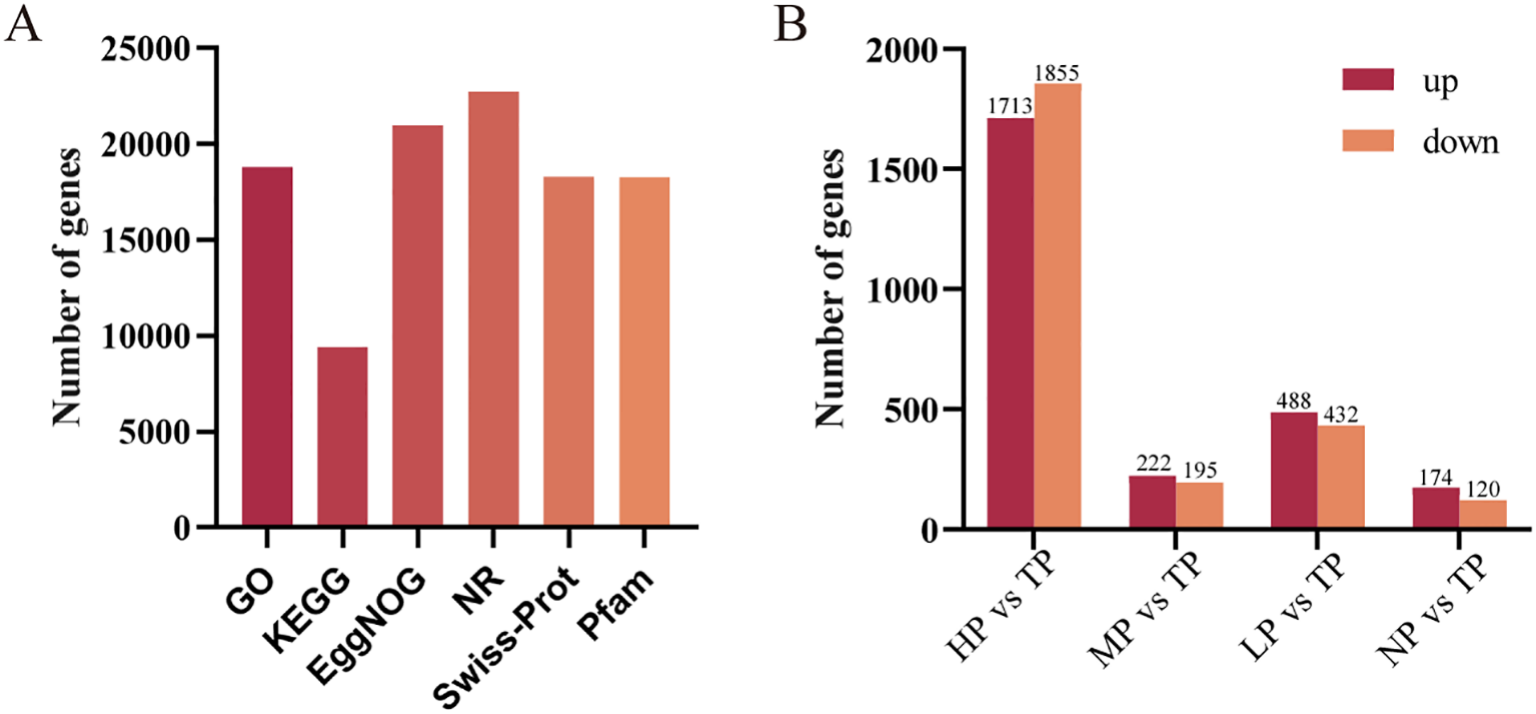
Transcriptome sequencing and gene expression analysis. **(A)** Functional annotation of unigenes; **(B)** Number of DEGs in reponse to the induction of phosphorus deficiency in four comparative combinations. TP, HP, MP, LP, and NP denote total, high, medium, low and no phosphorus, respectively.

By homologous sequence similarity alignment, a total of 25,286 coding genes were successfully annotated in the EggNOG, Swiss-Prot, GO, Pfam, KEGG, and NR databases, accounting for 96.56% of the total 26186 splicing unigenes. Among the successfully annotated genes, 20,959 unigenes (84.89%) were annotated in the EggNOG database, 18,294 unigenes (74.07%) in the Swiss-Prot database, 18,829 unigenes (78.72%) in the GO database, 18,289 unigenes (74.02%) in the Pfam database, 9,417 unigenes (37.74%) in the KEGG database, and 22,712 unigenes (96.56%) in the NR database (**Figure 2A**; **Supplementary Table S2**). Among them, 7,744 unigenes were matched to the known sequences in multiple databases.

Through differentially expressed gene (DEG) analysis, the DEGs in response to phosphorus deficiency in *D. officinale* were identified (**Figure 2B**; **Supplementary Table S4**). In total, 4,049 DEGs were identified. Among these, 1,713, 222, 488 and 174 DEGs were up-regulated in the HP vs. TP, MP vs. TP, LP vs. TP and NP vs. TP comparisons, respectively.

**Figure 3:**
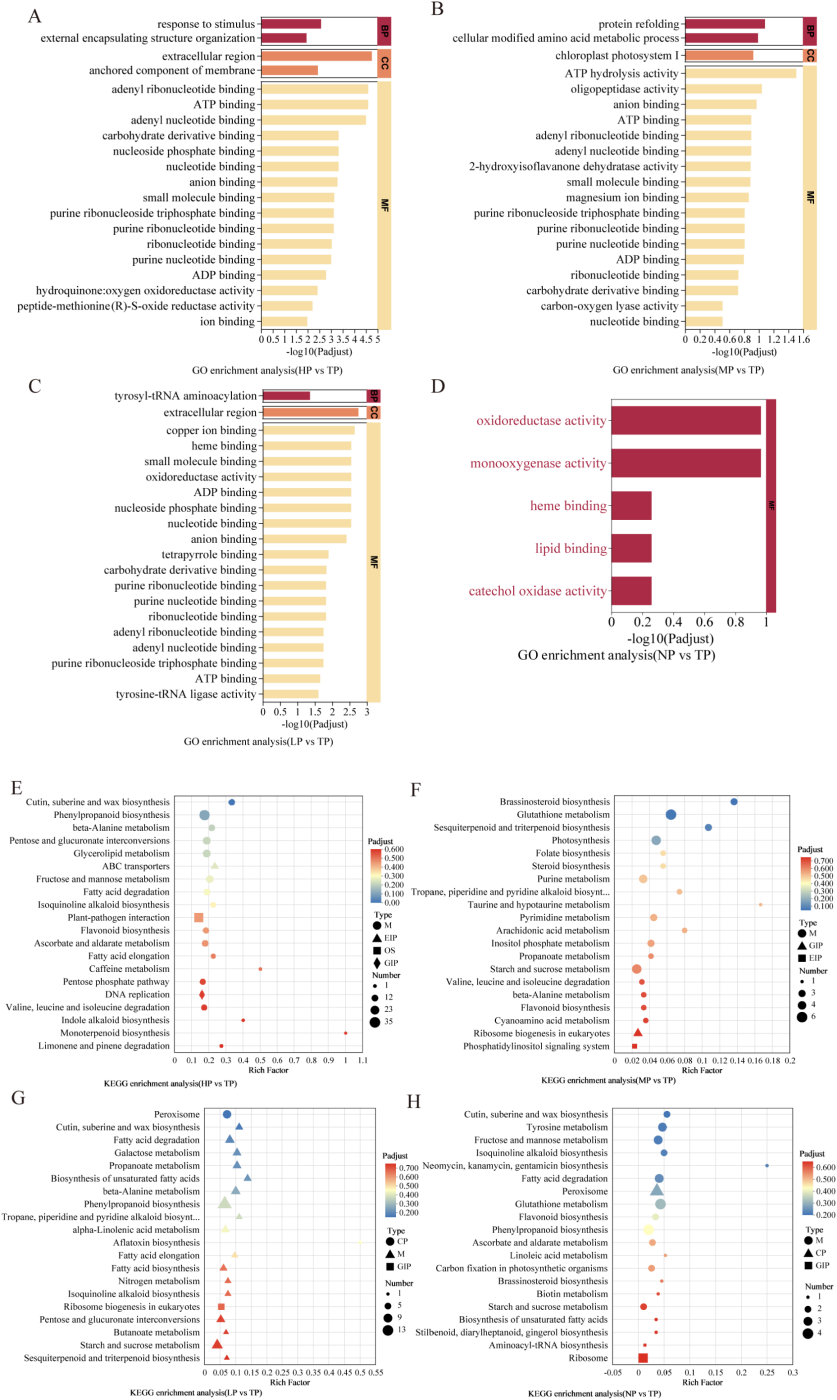
GO and KEGG pathway enrichment analysis of the DEGs in four comparative groups. **(A-D)** GO enrichment analysis of the DEGs. The visualization presents the significantly enriched GO semantic categories (y-axis) against the magnitude of their enrichment (x-axis), which is expressed as -log_10_(P-adjust). Therefore, the length of each bar directly reflects the statistical significance of the enrichment for that particular GO term. **(E-H)** KEGG pathway enrichment analysis of the DEGs is summarized in this visualization. Pathways are arranged along the y-axis. For each pathway, its enrichment significance (rich factor) is indicated on the x-axis, and the scale of the associated gene set is represented by the size of the circle. Log_2_FC|≥1 and an adjusted *P*-value<0.05 was defined as the cutoff to select the DEGs. TP, HP, MP, LP, and NP denote total, high, medium, low and no phosphorus, respectively.

Gene Ontology (GO) and Kyoto Encyclopedia of Genes and Genomes (KEGG) enrichment analysis were introduced to predict the potential function of DEGs. GO enrichment analysis was performed to annotate the DEGs in the LP vs TP group. In the cellular component (CC) category, extracellular region was the most enriched subcategories. In the biological process (BP) category, tyrosyl-tRNA aminoacylation peaked at all the function classifications. In the molecular function (MF) category, copper ion binding, heme binding, small molecule binding and oxidoreductase activity were the top four enrichments (**Figure 3**; **Supplementary Table S5-8**).

By KEGG enrichment analysis, cutin, suberin, and wax biosynthesis, phenylpropanoid biosynthesis, β-alanine metabolism, pentose and glucuronate interconversions represented the top four pathways in the HP vs. TP comparative group (**Supplementary Table S9**). In the NP vs. TP comparative group, the DEGs were preferentially enriched in the pathways of cutin, suberin, and wax biosynthesis, tyrosine metabolism, fructose and mannose metabolism, and isoquinoline alkaloid biosynthesis (**Supplementary Table S10**). In the MP vs. TP group, pathways were predominantly enriched in brassinosteroid biosynthesis, glutathione metabolism, photosynthesis, and sesquiterpenoid and triterpenoid biosynthesis (**Supplementary Table S11**). The LP vs. TP groups exhibited significant pathway enrichment in peroxisome, cutin, suberin and wax biosynthesis, and fatty acid degradation. Notably, the biosynthesis of tropane, piperidine, and pyridine alkaloids (terpenoid alkaloids) exhibited higher rich factors compared to other groups. This finding indicates that under phosphorus deficiency, enzyme-coding genes associated with terpenoid alkaloid synthesis become substantially enriched (**Supplementary Table S12**).

### Mining of the candidate transcription factors in response to low phosphorus stress

**Figure 4:**
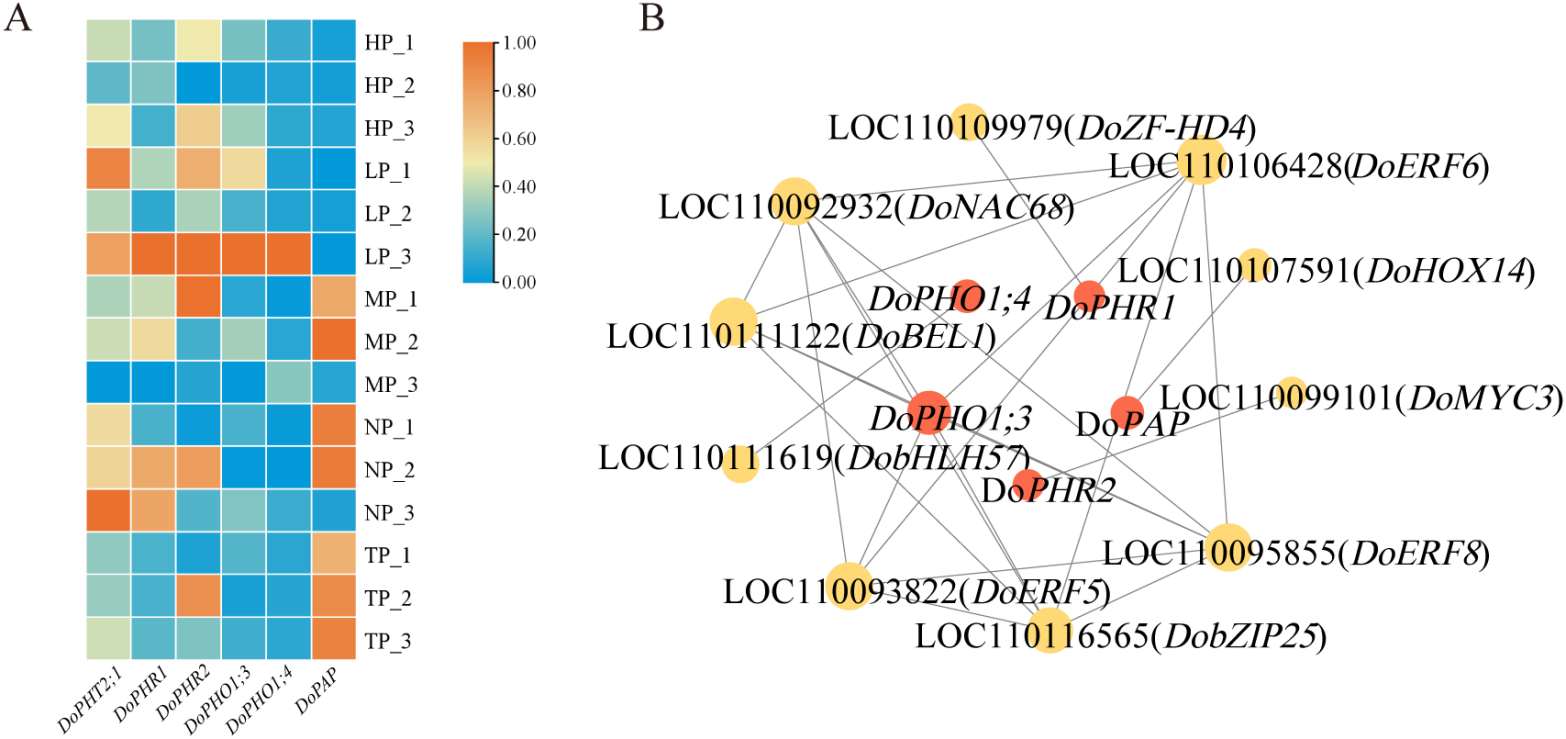
Construction of co-expression network to identify the candidate TFs involved in LP stress and heatmap of differentially expressed genes related to phosphorus (P)-transport in response to LP stress. **(A)** Phosphorus transporter gene expression profiles. **(B)** Network analysis of transcriptional regulators and phosphorus transporter genes. PHT, phosphate transporter; PHO, phosphate; PHR, phosphate starvation response; PAP, purple acid phosphatase. TP, HP, MP, LP and NP denote total, high, medium, low and no phosphorus, respectively.

To identify candidate transcription factors (TFs) responsive to low-phosphorus (LP) stress, a gene co-expression network was constructed. As the core drivers of phosphorus uptake and allocation, key phosphorus transport and signaling genes serve as ideal entry points for discovering their upstream regulators. Based on functional annotation,six such “bait” genes were selected, each representing a pivotal component of the conserved plant phosphate response network: *DoPHT2* (a phosphate transporter central to Pi uptake, LOC110101372), *DoPHO1;3* (LOC110106399) and *DoPHO1;4* (mediators of phosphate allocation from roots to shoots, LOC110098888), *DoPHR1* (LOC110110494) and *DoPHR2* (master transcription factors of phosphate starvation response, LOC110113993), and *DoPAP* (a purple acid phosphatase involved in phosphorus deficiency adaptation, LOC110111096) (**Figure 4A**; **Supplementary Table S13**). These six bait genes, together with 194 TFs identified from the DEGs, were then subjected to co-expression correlation analysis. A co-expression network containing 15 nodes and 25 links was constructed with the Pearson correlation coefficients >0.90 as the cutoff. In total, 10 candidate TFs were identified that exhibited strong correlations with five of the above six P-transport genes (*DoPHO1;3*, *DoPHO1;4*, *DoPHR1*, *DoPHR2*, and *DoPAP*). Among the 10 correlated TFs, *DoNAC68* (gene-LOC110092932) and *DoMYC3* (gene-LOC110099101) exhibited the highest correlations with the above five P-transport genes, followed by *DoERF5* (LOC110093822), *DoERF8* (LOC110095855), *DobHLH57* (LOC110111619), *DoERF6* (LOC110106428), *DobZIP25* (LOC110116565), *DoBEL1* (LOC110111122), *DoHOX14* (LOC110107591) and *DoZF-HD4* (LOC110109979), which were named based on their homologs in the genome of *Arabidopsis thaliana* (**Figure 4B**; **Supplementary Table S14**).

### Mining of the candidate transcription factors involved in dendrobine biosynthesis

**Figure 5:**
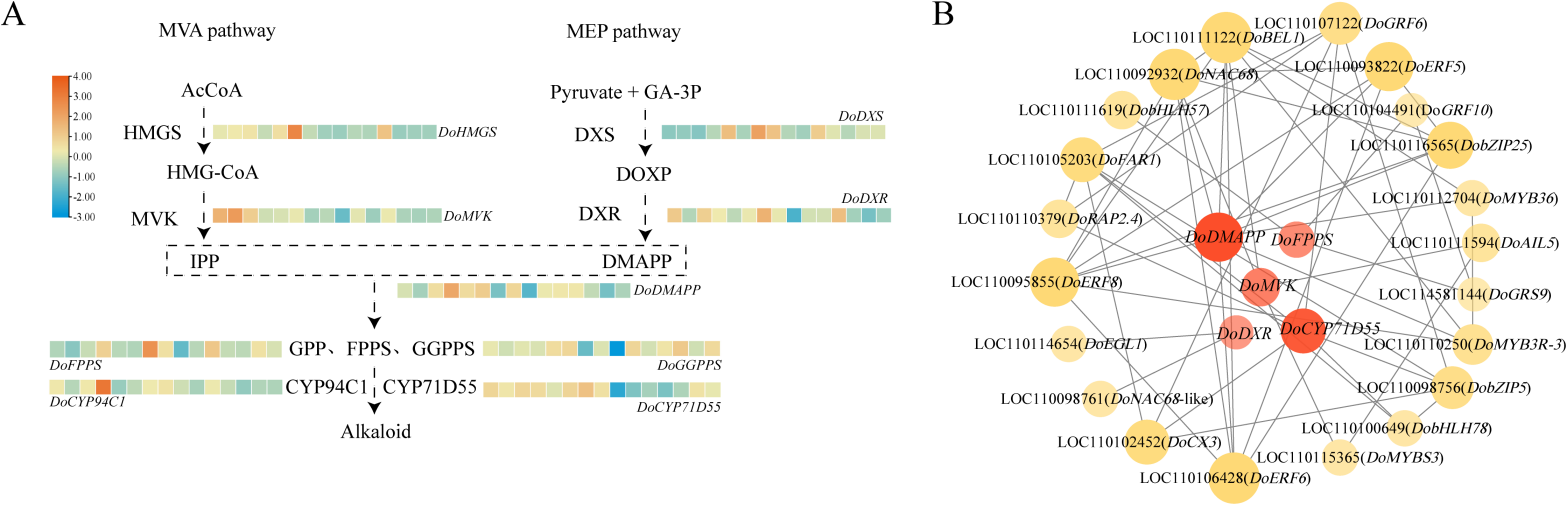
Candidate TF mining via co−expression network and expression analysis of DEGs involved in dendrobine biosynthesis. **(A)** Phosphorus−induced expression profiles of dendrobine synthase genes. **(B)** Construction of a co−expression network between candidate TFs and dendrobine biosynthetic genes. TP, HP, MP, LP and NP denote total, high, medium, low, and no phosphorus, respectively.

To identify candidate TFs involved in regulating dendrobine biosynthesis, a co-expression network containing 26 nodes and 63 links was constructed using 194 selected TFs and 5 known dendrobine biosynthetic genes including *DoDMAPP* (LOC110095726), *DoFPPS* (LOC110096432), *DoMVK* (LOC110113415), *DoDXR* (LOC110096522) and *DoCYP71D55* (LOC110114012). Pearson correlation coefficients >0.9 and an adjusted *P*-value <0.05 were used as cutoffs (**Figure 5A**; **Supplementary Table S15**). In this co-expression network, *DoRAP2.4* (LOC110110379) and *DoGRF10* (LOC110104491) exhibited the highest Pearson correlation coefficients reaching to 0.983, followed by *DoFAR1* (LOC110105203), *DoGRF6* (LOC110107122), *DoNAC68* (LOC110092932), *DoERF8* (LOC110095855), and *DoBEL1* (LOC110111122) with the Pearson correlation coefficients greater than 0.95 (**Figure 5B**; **Supplementary Table S16**). These TFs were thought to be the potential regulators of dendrobine biosynthesis in *D. officinale*.

### Experimental validation of differential expressed TFs by qRT-PCR analysis

**Figure 6:**
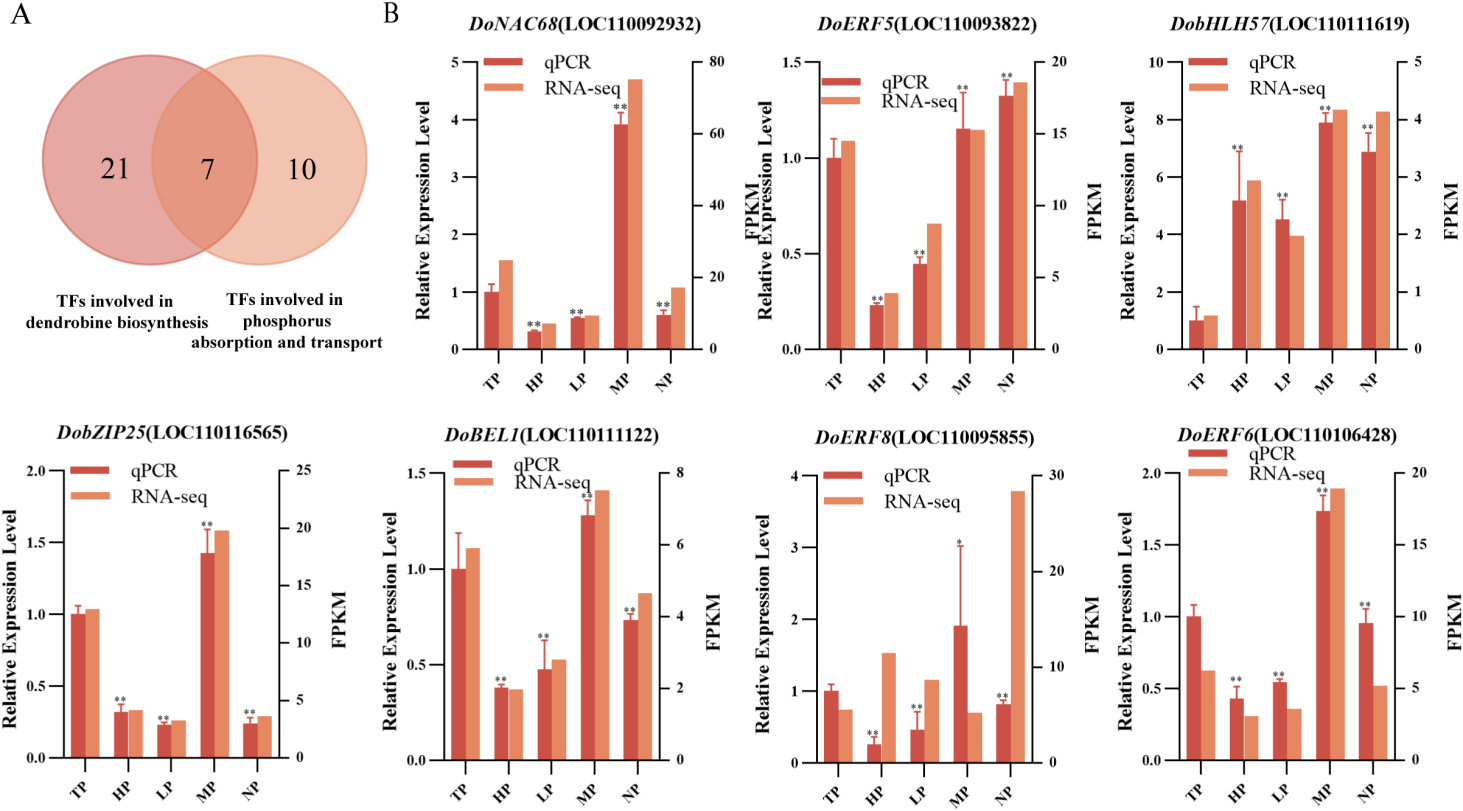
Experimental validation of the candidate TFs co-regulating LP stress and dendrobine biosynthesis. **(A)** Venn diagram of TFs from two co-expression networks. **(B)** qRT−PCR validation of the seven candidate TFs in (*D*) *officinale*, compared with transcriptome−derived expression levels. The TP group was taken as the reference. Data are presented as mean ± SD (*n* = 3). Significant differences were determined by Student’s t-test (*P < 0.05; **P < 0.01). TP, HP, MP, LP and NP denote total, high, medium, low, and no phosphorus, respectively.

To identify potential co-regulators involved in both dendrobine biosynthesis and LP stress response, this study conducted an integrative analysis combining 10 candidate TFs implicated in the regulation of dendrobine biosynthesis with 21 potential candidate TFs responsive to LP stress (**Figure 6A**). This analysis revealed seven overlapping candidate TFs: *DoNAC68* (LOC110092932), *DoERF5* (LOC110093822), *DobHLH57* (LOC110111619), *DobZIP25* (LOC110116565), *DoBEL1* (LOC110111122), *DoERF8* (LOC110095855) and *DoERF6* (LOC110106428). The expression patterns of all seven candidate TFs were subsequently validated by qRT−PCR under a gradient of phosphorus concentrations. The results confirmed that their expression trends were highly consistent with the transcriptomic data, with correlation coefficients ranging from 0.92 to 0.99 (**Figure 6B**; **Supplementary Figure S2**). These results demonstrate the reliability of the transcriptomic data in mining the candidate TFs involved in modulating dendrobine biosynthesis under phosphate deficiency in *D. officinale*.

## Discussion

Phosphorus acts as an essential nutrient in regulating plant growth, development, and metabolism (Gu et al., 2016). In wild environment, *D. officinale* prefers to grow as epiphytes on tree bark and rocks. This species characteristic enables the *D. officinale* to adapt to the poor nutrient environment (Xiang et al., 2016; Hou et al., 2017; Ren et al., 2020; He et al., 2024). These findings prompted an investigation into whether LP stress genuinely enhances the quality of *D. officinale* by promoting dendrobine accumulation in its stems (**Figure 1**). In *Anisodus tanguticus*, phosphorus deficiency enhances arbuscular mycorrhiza-mediated coordination of carbon-nitrogen-phosphorus metabolism, thereby promoting alkaloid accumulation (Zhang et al., 2025). LP stress has been validated to improve the tanshinone biosynthesis in *S. miltiorrhiza* hairy roots (Zheng et al., 2023). In summary, the judicious application of phosphorus during the production of traditional Chinese medicinal materials is critical, as it not only ensures product quality but also minimizes phosphate fertilizer usage, thereby alleviating environmental discharge pressures.

Co-expression network analysis is thought to be a reliable strategy for identifying candidate TFs in plant species (Liu et al., 2022; Han et al., 2024; Zhang et al., 2024). For example, integration of transcriptome data and co-expression network analysis identified the core transcription factor *MabHLH355* in banana, which regulates reactive oxygen species (ROS) scavenging under cold stress ( (Lin et al., 2024). Under low−nitrogen stress, six transcription factors implicated in the biosynthesis of tanshinones and salvianolic acids were identified in *S. miltiorrhiza* (Cheng et al., 2025b). By gene co-expression network analysis, 4 important TFs including *PpZAT6*, *PpPMZ*, *PpMYB-like*, and *PpONAC077* in association with the terpenoid metabolites biosynthesis were identified in *Poa pratensis* (Poirier et al., 2022). In this study, based on transcriptomic sequencing data in combination with co-expression correlation analyses, 21 TFs related to dendrobine biosynthesis and 10 TFs involved in phosphorus absorption and transport were identified, respectively. By integrating the above two co-expression networks, seven TFs are identified: *DoNAC68* (LOC110092932), *DoERF5* (LOC110093822), *DobHLH57* (LOC110111619), *DobZIP25* (LOC110116565), *DoBEL1* (LOC110111122), *DoERF8* (LOC110095855) and *DoERF6* (LOC110106428). As the homologous gene of *DoERF8* (LOC110095855), *AtERF8* functions as a transcriptional repressor that modulates light-responsive anthocyanin biosynthesis in *A. thaliana* (Koyama and Sato, 2018; Isidra-Arellano et al., 2021; Ren et al., 2022). *DoGRF6* (LOC110107122) exhibits a high sequence similarity with *ClNAC68* in *Citrullus lanatus*, which has been verified to regulate sugar accumulation during maturation stage in watermelon (Pertea et al., 2015). *DoBEL1* (LOC110111122) is a homolog of *MaBEL1* in banana, and it was verified to activate the expression of genes involved in starch degradation thereby promoting fruit ripening (Song et al., 2023). Although the functions of these genes still require experimental validation in *D. officinale*, they provide critical clues for elucidating the underlying biological functions of the seven candidate transcription factors identified in this study.

Phosphate transporters (PHTs), acting as the high-affinity phosphate transporters, localize to the plasma membrane and they determine phosphorus uptake and redistribution via plant root (Paz-Ares et al., 2022). Among them, PHT1;1 and PHT1;4 exhibit a root-specific upregulation expression pattern in response to LP stress (Tao et al., 2024). PHO1 members were generally thought to mediate phosphorus transport from roots to shoots in plant species (Isidra-Arellano et al., 2021). Therefore, to identify candidate TFs responsive to LP stress, *DoPHO1;3*, *DoPHO1;4*, *DoPHR1*, *DoPHR2*, and *DoPAP* were employed as ‘baits’ to construct a co-expression network to prey the candidate TFs among the 194 differentially expressed TFs. Ultimately, 10 TFs including *DoNAC68, DoMYC3, DoERF5*, *DoERF8*, *DobHLH57*, *DoERF6*, *DobZIP25*, *DoBEL1*, *DoHOX14*, and *DoZF-HD4* were strongly correlated (r > 0.9) with the above 6 phosphorus transport-related genes (**Figure 4B**; **Supplementary Table S14**). In apple, MdMYB306 interacts with MdMYB17 and MdbHLH33 to inhibit anthocyanin synthesis (Wang et al., 2022). The CUL3-BPM E3 ubiquitin ligase AtPUB10 has been reported to regulate jasmon acid signaling by modulating the stability of AtMYC2, AtMYC3, and AtMYC4 proteins in *A.thaliana* (Chico et al., 2020). In *Catharanthus roseus*, *CrERF5*, an AP2/ERF transcription factor, modulates the production of bisindole alkaloids through activating the expression of tryptophan decarboxylase (TDC) gene (Teng et al., 2025). AtbHLH57 has been validated to interact with AtODR1 (REVERSAL OF RDO5 1) to regulate ABA synthesis in *A.thaliana* (Liu et al., 2020a). The ABA pathway integrates environmental signals through a signal transduction network to regulate metabolic processes within plants, enabling them to adapt to adverse conditions. Studies on *S. miltiorrhiza* reveal that plants adapt to environmental changes by improving anthocyanin synthesis under LP stress (Tao et al., 2024). Therefore, elucidating how these 10 TFs participate in phosphate uptake and secondary metabolite synthesis in *D. officinale*, with reference to previous reports, is worth exploring.

## Conclusions

The present study has employed transcriptome sequencing data of *D. officinale* in combination with gene co-expression network analysis to identify candidate TFs involved in regulation of dendrobine biosynthesis under LP stress. In total, 7 candidate TFs are mined. qRT-PCR analysis showed that their expression levels are drastically induced under LP stress compared to the Mock (TP), and this induction trend is consistent with the transcriptome sequencing data. In general, this study offers many valuable candidate TFs for elucidating the underlying molecular mechanism on modulating dendrobine biosynthesis under LP stress. These findings will promote scientific production with the condition of low phosphorus consumption in *D. officinale*.

## Data Availability

The datasets presented in this study can be found in online repositories. The names of the repository/repositories and accession number(s) can be found below: https://www.ncbi.nlm.nih.gov/, PRJNA1400121.

## References

[B1] Cakova V. Bonte F. Lobstein A. (2017). Dendrobium: sources of active ingredients to treat age-related pathologies. Aging Dis. 8, 827–849. doi: 10.14336/AD.2017.0214, PMID: 29344419 PMC5758354

[B2] Chen J. Zhao Y. Chen X. Li Y. Kang L. Liu Y. (2025). The antiviral properties of flavonoids. Clin. Trad. Med. Pharmacol. 6, 200192. doi: 10.1016/j.ctmp.2024.200192, PMID: 41743167

[B3] Chen S. Zhou Y. Chen Y. Gu J. (2018). Fastp: an ultra-fast all-in-one FASTQ preprocessor. Bioinformatics. 34, i884–i890. doi: 10.1093/bioinformatics/bty560, PMID: 30423086 PMC6129281

[B4] Cheng Y. Gui S. Hao S. Li X. Zhuang C. Shi Y. . (2025a). Mining the candidate transcription factors modulating tanshinones’ and phenolic acids’ biosynthesis under low nitrogen stress in *Salvia miltiorrhiza*. Int. J. Mol. Sci. 26. doi: 10.3390/ijms26041774, PMID: 40004237 PMC11855394

[B5] Cheng C. Pan P. Wu Y. (2025b). Dietary sarcodia suieae hydrocolloid supplementation elevates quercetin levels and modulates metabolic and immune pathways in the hepatopancreas of macrobrachium rosenbergii: insights from integrated omics analyses. Dev. Comp. Immunol. 173, 105511. doi: 10.1016/j.dci.2025.105511, PMID: 41223956

[B6] Chico J. M. Lechner E. Fernandez-Barbero G. Canibano E. García-Casado G. Rubio V. . (2020). CUL3(BPM) E3 ubiquitin ligases regulate MYC2, MYC3, and MYC4 stability and JA responses. Proc. Natl. Acad. Sci. U. S. A. 117, 6205–6215. doi: 10.1073/pnas.1912199117, PMID: 32123086 PMC7084108

[B7] Gong D. Y. Chen X. Y. Guo S. X. Wang B. C. Li B. (2021). Recent advances and new insights in biosynthesis of dendrobine and sesquiterpenes. Appl. Microbiol. Biotechnol. 105, 6597–6606. doi: 10.1007/s00253-021-11534-1, PMID: 34463801

[B8] Gu R. Chen F. Long L. Cai H. Liu Z. Yang J. . (2016). Enhancing phosphorus uptake efficiency through QTL-based selection for root system architecture in maize. J. Genet. Genomics 43, 663–672. doi: 10.1016/j.jgg.2016.11.002, PMID: 27889500

[B9] Guo L. Yang Y. Pu Y. Mao S. Nie Y. Liu Y. . (2024). *Dendrobium officinale* kimura & migo polysaccharide and its multilayer emulsion protect skin photoaging. J. Ethnopharmacol. 318, 116974. doi: 10.1016/j.jep.2023.116974, PMID: 37517571

[B10] Han C. Shi C. Liu L. Han J. Yang Q. Wang Y. . (2024). Majorbio cloud 2024: update single-cell and multiomics workflows. Imeta 3. doi: 10.1002/imt2.217, PMID: 39135689 PMC11316920

[B11] Hao X. Pu Z. Cao G. You D. Zhou Y. Deng C. . (2020). Tanshinone and salvianolic acid biosynthesis are regulated by *SmMYB98* in *Salvia miltiorrhiza* hairy roots. J. Adv. Res. 23, 1–12. doi: 10.1016/j.jare.2020.01.012, PMID: 32071787 PMC7016019

[B12] He W. Guan N. Huang R. Huang X. Qu L. Zhong Z. . (2024). The effects of traditional Chinese medicine on growth factors. Clin. Trad. Med. Pharmacol. 5, 200131. doi: 10.1016/j.ctmp.2024.200131, PMID: 41743167

[B13] Hou B. Luo J. Zhang Y. Niu Z. Xue Q. Ding X. (2017). Iteration expansion and regional evolution: phylogeography of *Dendrobium officinale* and four related taxa in southern China. Sci. Rep. 7, 43525. doi: 10.1038/srep43525, PMID: 28262789 PMC5337965

[B14] Huang P. Li Z. Wang H. Huang J. Tan G. Fu Y. . (2024). A genome assembly of decaploid houttuynia cordata provides insights into the evolution of houttuynia and the biosynthesis of alkaloids. Hortic. Res. 11, uhae203. doi: 10.1093/hr/uhae203, PMID: 39308792 PMC11415239

[B15] Huang C. Yu J. Da J. Dong R. Dai L. Yang Y. . (2023). *Dendrobium officinale* kimura & migo polysaccharide inhibits hyperglycaemia-induced kidney fibrosis via the miRNA-34a-5p/SIRT1 signalling pathway. J. Ethnopharmacol. 313, 116601. doi: 10.1016/j.jep.2023.116601, PMID: 37146843

[B16] Inubushi Y. Sasaki Y. Tsuda Y. Yasui B. Konita T. Matsumoto J. . (1963). Structure of dendrobine. Yakugaku. Zasshi. 83, 1184–1186. doi: 10.1248/yakushi1947.83.12_1184, PMID: 14094221

[B17] Isidra-Arellano M. C. Delaux P. E. R. M. Valdés-López O. (2021). The phosphate starvation response system: its role in the regulation of plant-microbe interactions. Plant Cell Physiol. 62, 392–400. doi: 10.1093/pcp/pcab016, PMID: 33515263

[B18] Kim D. Langmead B. Salzberg S. L. (2015). Hisat: a fast spliced aligner with low memory requirements. Nat. Methods 12, 357–360. doi: 10.1038/nmeth.3317, PMID: 25751142 PMC4655817

[B19] Koyama T. Sato F. (2018). The function of *ETHYLENE RESPONSE FACTOR* genes in the light-induced anthocyanin production of arabidopsis thaliana leaves. Plant Biotechnol. 35, 87–91. doi: 10.5511/plantbiotechnology.18.0122b, PMID: 31275041 PMC6543729

[B20] Li K. Deng X. Wu Y. Li J. Huang M. Liu C. . (2024). Identification and validation of reference genes for qrt-pcr analyses under different experimental conditions in dendrobium nobile. Med. Plant Biol. 4. doi: 10.48130/mpb-0024-0031 36657231

[B21] Li Q. Ding G. Li B. Guo S. X. (2017). Transcriptome analysis of genes involved in dendrobine biosynthesis in *Dendrobium nobile* Lindl. Infected with mycorrhizal fungus MF23 (*Mycena* sp.). Sci. Rep. 7. doi: 10.1038/s41598-017-00445-9, PMID: 28331229 PMC5428410

[B22] Li D. Lu P. Li F. Qi Q. Lai Z. Li J. . (2025). Sweet transporters in dendrobium species: molecular insights into the regulation of polysaccharide biosynthesis. Med. Plant Biol. 4. doi: 10.48130/mpb-0025-0028

[B23] Lin S. Song W. Gan L. Wei W. Shan W. Kuang J. . (2024). Low temperature downregulates MabHLH355 and its associated target genes responsible for scavenging ros in banana peel under cold stress. Posthar. Biol. Technol. 213, 112956. doi: 10.1016/j.postharvbio.2024.112956, PMID: 41743167

[B24] Liu L. Xiang H. Shen H. Dong Y. Sun X. Cai Y. . (2021). Effects of low phosphorus stress on the main active ingredients and antioxidant activities of *Dendrobium officinale*. Ind. Crop Prod. 173. doi: 10.1016/j.indcrop.2021.114095, PMID: 41743167

[B25] Liu L. Xiang H. Song J. Shen H. Sun X. Tian L. . (2022). Genome - wide analysis of *DoSPX* genes and the function of *DoSPX4* in low phosphorus response in *Dendrobium officinale*. Front. Plant Sci. 13. doi: 10.3389/fpls.2022.943788, PMID: 35898219 PMC9313600

[B26] Liu Y. Yang L. Zhang Y. Liu X. Wu Z. Gilbert R. G. . (2020a). *Dendrobium officinale* polysaccharide ameliorates diabetic hepatic glucose metabolism via glucagon-mediated signaling pathways and modifying liver-glycogen structure. J. Ethnopharmacol. 248, 112308. doi: 10.1016/j.jep.2019.112308, PMID: 31622745

[B27] Liu F. Zhang H. Ding L. Soppe W. J. J. Xiang Y. (2020b). *REVERSAL of RDO5* 1, a homolog of rice seed dormancy4, interacts with bHLH57 and controls ABA biosynthesis and seed dormancy in *Arabidopsis*. Plant Cell. 32, 1933–1948. doi: 10.1105/tpc.20.00026, PMID: 32213638 PMC7268807

[B28] Love M. I. Huber W. Anders S. (2014). Moderated estimation of fold change and dispersion for rna-seq data with deseq2. Genome Biol. 15, 550. doi: 10.1186/s13059-014-0550-8, PMID: 25516281 PMC4302049

[B29] Okoro N. O. Odiba A. S. Yu Q. He B. Liao G. Jin C. . (2023). Polysaccharides extracted from dendrobium officinale grown in different environments elicit varying health benefits in caenorhabditis elegans. Nutrients 15. doi: 10.3390/nu15122641, PMID: 37375545 PMC10301227

[B30] Paz-Ares J. Puga M. I. Rojas-Triana M. Martinez-Hevia I. Diaz S. Poza-Carrion C. . (2022). Plant adaptation to low phosphorus availability: core signaling, crosstalks, and applied implications. Mol. Plant 15, 104–124. doi: 10.1016/j.molp.2021.12.005, PMID: 34954444

[B31] Pertea M. Pertea G. M. Antonescu C. M. Chang T. C. Mendell J. T. Salzberg S. L. (2015). StringTie enables improved reconstruction of a transcriptome from RNA-seq reads. Nature Biotechnology 33, 290–295. doi: 10.1038/nbt.3122, PMID: 25690850 PMC4643835

[B32] Poirier Y. Jaskolowski A. Clua J. (2022). Phosphate acquisition and metabolism in plants. Curr. Biol. 32, R623–R629. doi: 10.1016/j.cub.2022.03.073, PMID: 35728542

[B33] Ren Z. Ji X. Jiao Z. Luo Y. Zhang G. Q. Tao S. . (2020). Functional analysis of a novel c-glycosyltransferase in the orchid *Dendrobium catenatum*. Hortic. Res. 7, 111. doi: 10.1038/s41438-020-0330-4, PMID: 32637139 PMC7326982

[B34] Ren Y. Yu G. Shi C. Liu L. Guo Q. Han C. . (2022). Majorbio cloud: a one-stop, comprehensive bioinformatic platform for multiomics analyses. Imeta 1. doi: 10.1002/imt2.12, PMID: 38868573 PMC10989754

[B35] Shi M. Luo X. Ju G. Li L. Huang S. Zhang T. . (2016). Enhanced diterpene tanshinone accumulation and bioactivity of transgenic *Salvia miltiorrhiza* hairy roots by pathway engineering. J. Agric. Food. Chem. 64, 2523–2530. doi: 10.1021/acs.jafc.5b04697, PMID: 26753746

[B36] Song Z. Zhu X. Lai X. Chen H. Wang L. Yao Y. . (2023). Mabel1 regulates banana fruit ripening by activating cell wall and starch degradation-related genes. J. Integr. Plant Biol. 65, 2036–2055. doi: 10.1111/jipb.13506, PMID: 37177912

[B37] Suttipanta N. Pattanaik S. Kulshrestha M. Patra B. Singh S. K. Yuan L. . (2011). The transcription factor *CrWRKY1* positively regulates the terpenoid indole alkaloid biosynthesis in *Catharanthus roseus*. Plant Physiol. 157, 2081–2093. doi: 10.1104/pp.111.181834, PMID: 21988879 PMC3327198

[B38] Tao H. Gao F. Li L. He Y. Zhang X. Wang M. . (2024). *WRKY33* negatively regulates anthocyanin biosynthesis and cooperates with *PHR1* to mediate acclimation to phosphate starvation. Plant Commun. 5. doi: 10.1016/j.xplc.2024.100821, PMID: 38229439 PMC11121177

[B39] Teng K. Zhao N. Xie Y. Li R. Li J. (2025). An AP2/ERF transcription factor controls generation of the twin-seedling rice. J. Adv. Res. 76, 33–43. doi: 10.1016/j.jare.2024.12.013, PMID: 39701377 PMC12793751

[B40] Udomsom N. Rai A. Suzuki H. Okuyama J. Imai R. Mori T.Mori T. . (2016). Function of AP2/ERF transcription factors involved in the regulation of specialized metabolism in *Ophiorrhiza pumila* revealed by transcriptomics and metabolomics. Front. Plant Sci. 7. doi: 10.3389/fpls.2016.01861, PMID: 28018397 PMC5145908

[B41] Wang Y. H. (2021). Traditional uses, chemical constituents, pharmacological activities, and toxicological effects of dendrobium leaves: a review. J. Ethnopharmacol. 270, 113851. doi: 10.1016/j.jep.2021.113851, PMID: 33485987

[B42] Wang H. Q. Jin M. Y. Paek K. Y. Piao X. C. Lian M. L. (2016). An efficient strategy for enhancement of bioactive compounds by protocorm-like body culture of *Dendrobium candidum*. Ind. Crop Prod. 84, 121–130. doi: 10.1016/j.indcrop.2016.02.001, PMID: 41743167

[B43] Wang S. Zhang Z. Li L. X. Wang H. B. Zhou H. Chen X. . (2022). Apple MdMYB306-like inhibits anthocyanin synthesis by directly interacting with MdMYB17 and MdbHLH33. Plant J. 110, 1021–1034. doi: 10.1111/tpj.15720, PMID: 35220614

[B44] Xiang X. G. Mi X. C. Zhou H. L. Li J. W. Chung S. W. Li D. . (2016). Biogeographical diversification of mainland Asian Dendrobium (Orchidaceae) and its implications for the historical dynamics of evergreen broad-leaved forests. J. Biogeogr. 43, 1310–1323. doi: 10.1111/jbi.12726, PMID: 41744481

[B45] Xu M. Wu C. Zhao L. Wang Y. Wang C. Zhou W. . (2020). WRKY transcription factor *OpWRKY1* acts as a negative regulator of camptothecin biosynthesis in *Ophiorrhiza pumila* hairy roots. Plant Cell Tissue Organ Cult. 142, 69–78. doi: 10.1007/s11240-020-01833-2, PMID: 41746348

[B46] Yang W. Chen D. Ji Q. Zheng J. Ma Y. Sun H. . (2023). Molecular mechanisms underlying the anticancer property of dendrobium in various systems of the human body: a review. Biomed. Pharmacother. 165, 115223. doi: 10.1016/j.biopha.2023.115223, PMID: 37523984

[B47] Zhang F. Li J. Wang T. Ma H. Zhou G. (2025). Integrated microbiological and metabolomic analyses reveal the mechanism by which P addition affects the quality of *Anisodus tanguticus*. Ind. Crop Prod. 228. doi: 10.1016/j.indcrop.2025.120956, PMID: 41743167

[B48] Zhang S. Qi X. Zhu R. Ye D. Shou M. Peng L. . (2024). Transcriptome analysis of *Salvia miltiorrhiza* under drought stress. Plants 13. doi: 10.3390/plants13020161, PMID: 38256715 PMC10819027

[B49] Zhang G. Xu Q. Bian C. Tsai W. Yeh C. Liu K. . (2016). The dendrobium catenatum lindl. Genome sequence provides insights into polysaccharide synthase, floral development and adaptive evolution. Sci. Rep. 6, 19029. doi: 10.1038/srep19029, PMID: 26754549 PMC4709516

[B50] Zhang Y. You S. Wang D. Zhao D. Zhang J. An Q. . (2022). Fermented dendrobium officinale polysaccharides protect UVA-induced photoaging of human skin fibroblasts. Food Sci. Nutr. 10, 1275–1288. doi: 10.1002/fsn3.2763, PMID: 35432966 PMC9007291

[B51] Zheng H. Fu X. Shao J. Tang Y. Yu M. Li L. . (2023). Transcriptional regulatory network of high-value active ingredients in medicinal plants. Trends Plant Sci. 28, 856. doi: 10.1016/j.tplants.2023.04.008, PMID: 36621413

[B52] Zhou W. Wang C. Hao X. Chen F. Huang Q. Liu T. . (2024). A chromosome-level genome assembly of anesthetic drug-producing anisodus acutangulus provides insights into its evolution and the biosynthesis of tropane alkaloids. Plant Commun. 5, 100680. doi: 10.1016/j.xplc.2023.100680, PMID: 37660252 PMC10811374

[B53] Zuo S. M. Yu H. D. Zhang W. Zhong Q. Chen W. Chen W. . (2020). Comparative metabolomic analysis of *Dendrobium officinale* under different cultivation substrates. Metabolites 10, 325. doi: 10.3390/metabo10080325, PMID: 32785071 PMC7465462

