# Supplementary material for: Mining the candidate transcription factors modulating dendrobine biosynthesis under phosphate deficiency in Dendrobium officinale Kimura & Migo
Source: Front Plant Sci. 2026 Mar 3;17:1784768. doi: 10.3389/fpls.2026.1784768 (PMC12992215; doi:10.3389/fpls.2026.1784768)
Supplement: Supplementary file 1 [file DataSheet1.docx]

Supplementary Figures


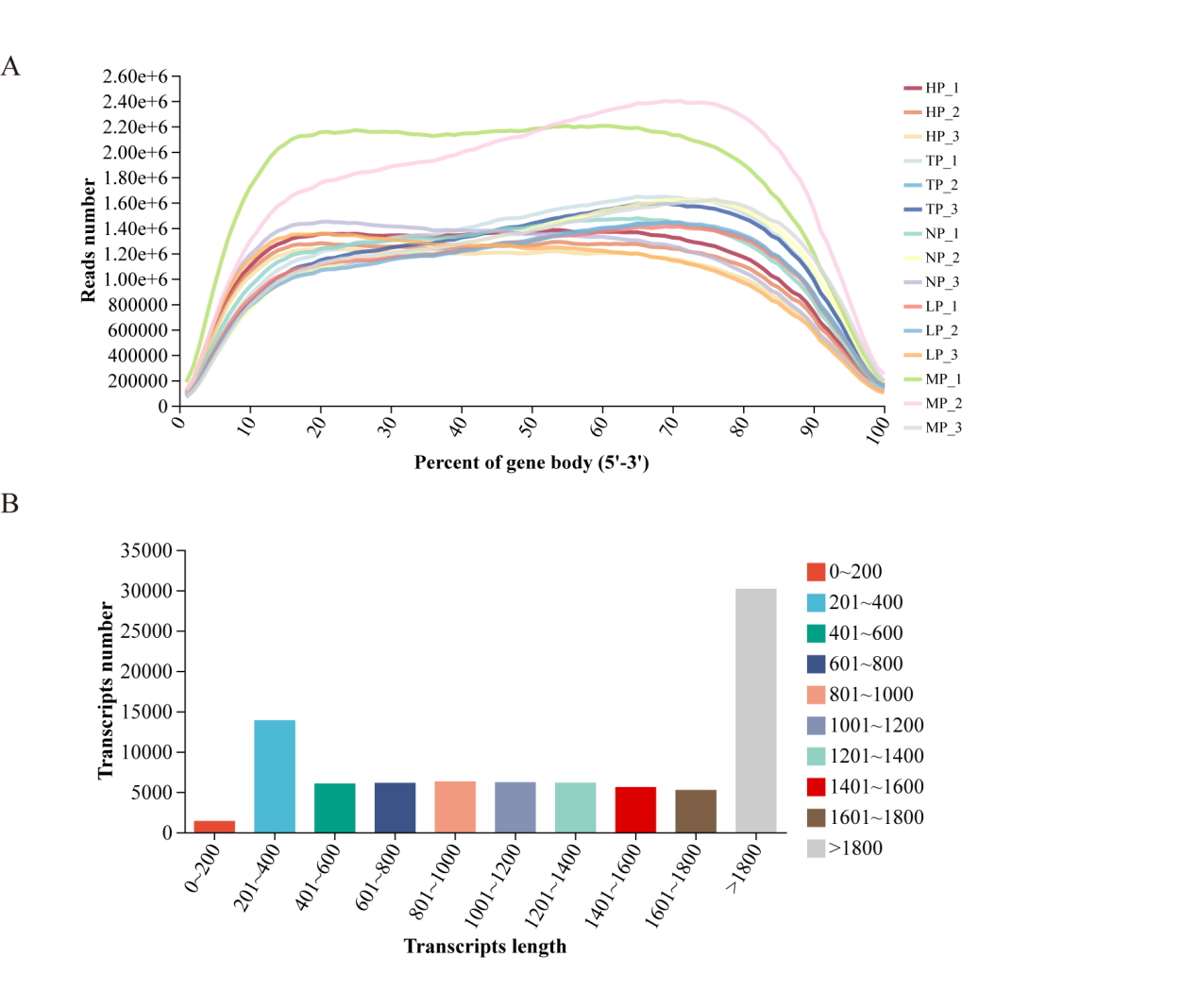


Figure S1. Transcriptome Assembly. (A) Sequencing coverage: The x-axis represents the relative position along normalized gene length (0% = 5’ terminus, 100% = 3’ terminus), while the y-axis indicates the total number of reads mapped to each corresponding genomic interval (bin) across all gene positions. (B) Transcript length distribution: The x-axis denotes transcript length intervals (bp), with the y-axis displaying corresponding transcript counts.


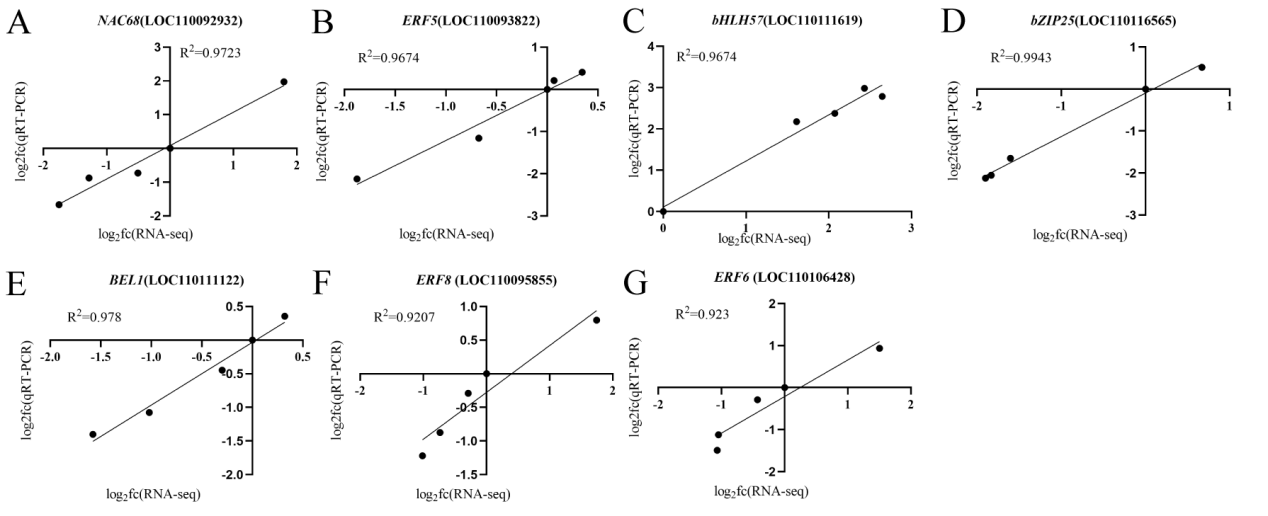
Figure S2. Correlation of expression profiles of 7 TFs obtained from qRT-PCR detection and RNA-seq.
